# Regulation of the MDM2-p53 nexus by a nuclear phosphoinositide and small heat shock protein complex

**DOI:** 10.1016/j.jbc.2025.110527

**Published:** 2025-07-26

**Authors:** Jeong Hyo Lee, Mo Chen, Tianmu Wen, Richard A. Anderson, Vincent L. Cryns

**Affiliations:** 1 University of Wisconsin Carbone Cancer Center, School of Medicine and Public Health, University of Wisconsin-Madison, Madison, Wisconsin, USA; 2 Department of Medicine, School of Medicine and Public Health, University of Wisconsin-Madison, Madison, Wisconsin, USA; 3 Department of Pharmacology, Joint Laboratory of Guangdong-Hong Kong Universities for Vascular Homeostasis and Diseases, SUSTech Homeostatic Medicine Institute, School of Medicine, Southern University of Science and Technology, Shenzhen, China

**Keywords:** MDM2, p53, PIP_2_, small heat shock protein, nucleus

## Abstract

The tumor suppressor p53 maintains genome stability in the setting of cellular stress and is frequently mutated in cancer. The stability of p53 is regulated by its interaction with the oncoprotein MDM2, a ubiquitin E3 ligase. Recently, nuclear phosphoinositides were reported to bind and stabilize p53. Here, we report that genotoxic stress induces the type I phosphatidylinositol phosphate kinase (PIPKIα) and its product phosphatidylinositol 4,5-bisphosphate (PIP_2_) to bind and regulate the stability and function of MDM2. Following genotoxic stress, nuclear PIPKIα binds to MDM2 to generate a complex of MDM2 and PIP_2_. PIP_2_ binding to MDM2 differentially regulates the recruitment of the small heat shock proteins (sHSPs) αB-crystallin (αBC) and HSP27 to the MDM2-PIP_2_ complex, acting as an on-off switch that regulates MDM2 stability, ubiquitination activity and interaction with p53. Our results demonstrate an unexpected role for nuclear phosphoinositides conferring specificity to the MDM2-PIP_2_-sHSPs association. Notably, the differential engagement of αBC and HSP27 reveals that sHSPs are not merely passive chaperones but play active, selective roles in fine-tuning MDM2 function and the MDM2-p53 nexus. These findings provide a previously unrecognized molecular framework for targeting this pathway in cancer.

Phosphatidylinositol-4,5-bisphosphate (PIP_2_) is the most abundant of the seven phosphoinositide lipid second messengers and plays a crucial role in regulating various cellular functions, including membrane trafficking, ion channel activity, and the actin cytoskeleton (1). PIP_2_ directly interacts with PIP_2_ effector proteins, controlling their activity and/or localization (2, 3). PIP_2_ is predominantly synthesized by the phosphorylation of phosphatidylinositol-4-phosphate (PI4P) and phosphatidylinositol-5-phosphate (PI5P) by type I (PI4P 5-kinases) and type II (PI5P 4-kinases) phosphatidylinositol phosphate kinases (PIPKs), respectively. The type I and type II kinases have α, β, and γ isoforms. PIPKIα, PIPKIβ, PIPKIγ, PIPKIIα and PIPKIIβ have reported nuclear activities (4). PIP_2_ is present in the nucleus as well as most membrane structures, including the plasma membrane and membranes of the endosome, Golgi, and endoplasmic reticulum. In the nucleus, PIP_2_ is located in regions distinct from the nuclear envelope in non-membranous nuclear speckles (5). Although several nuclear PIP_2_ effectors have been identified (2, 3, 5), the function of PIP_2_ in the nucleus is poorly understood.

One such nuclear PIP_2_ effector recently reported is the p53 tumor suppressor protein (wild-type and mutant forms) (3). Wild-type p53 defends the genome from various cellular stressors and is the most frequently mutated gene in human tumors (6, 7). p53 mutations result in the loss of wild-type p53 activities and gain of function, which promote cell survival and stress resistance to drive cancer initiation/progression (8, 9, 10, 11). Remarkably, PIPKIα and its product PIP_2_ interact with the C-terminal domain (CTD) of stress-activated wild-type p53 and mutant p53, resulting in the formation of highly stable p53-PIP_2_ complexes in the nucleus that are resistant to harsh denaturing conditions. These complexes then recruit the small heat shock proteins (sHSPs) αB-Crystallin (αBC) and HSP27, which bind and stabilize p53 (3). Moreover, the nuclear PI3K inositol polyphosphate multikinase (IPMK) and the 3-phosphatase phosphatase and tensin homolog (PTEN) bind p53 to catalyze the synthesis of p53-PIP_2_ to p53-phosphatidylinositol-3,4,5-triphosphate (PIP_3_) and the reverse reaction, respectively (12). p53-PIP_3_ recruits and activates Akt in the nucleus by a PIP_3_-dependent mechanism, providing a direct functional link between p53 and Akt in the nucleus. These findings indicate that phosphoinositides and PIP kinases dynamically and spatially regulate p53 stability and function in the nucleus.

Given the central role of the E3 ubiquitin-protein ligase, MDM2, in regulating p53 stability by ubiquitin-dependent proteasomal degradation of p53 (13, 14), we postulated that phosphoinositides and sHSPs might regulate the MDM2-p53 interaction by associating with p53 and/or MDM2. Indeed, both HSP90 and HSP70 bind mutant p53 and negatively regulate p53 degradation by MDM2 (15, 16, 17). Here, we report that MDM2, like p53, directly binds PIPKIα and its product PIP_2_, leading to the differential recruitment of the sHSPs αB-crystallin and HSP27 to MDM2, which regulates its stability, E3 ligase activity, and interaction with p53. Hence, both MDM2 and p53 are regulated by phosphoinositides and sHSPs, thereby providing exquisite temporal and spatial regulation of the MDM2-p53 nexus in the nucleus.

## Results

### PIP_2_ associates with MDM2 in the nucleus in response to stress

**Figure 1:**
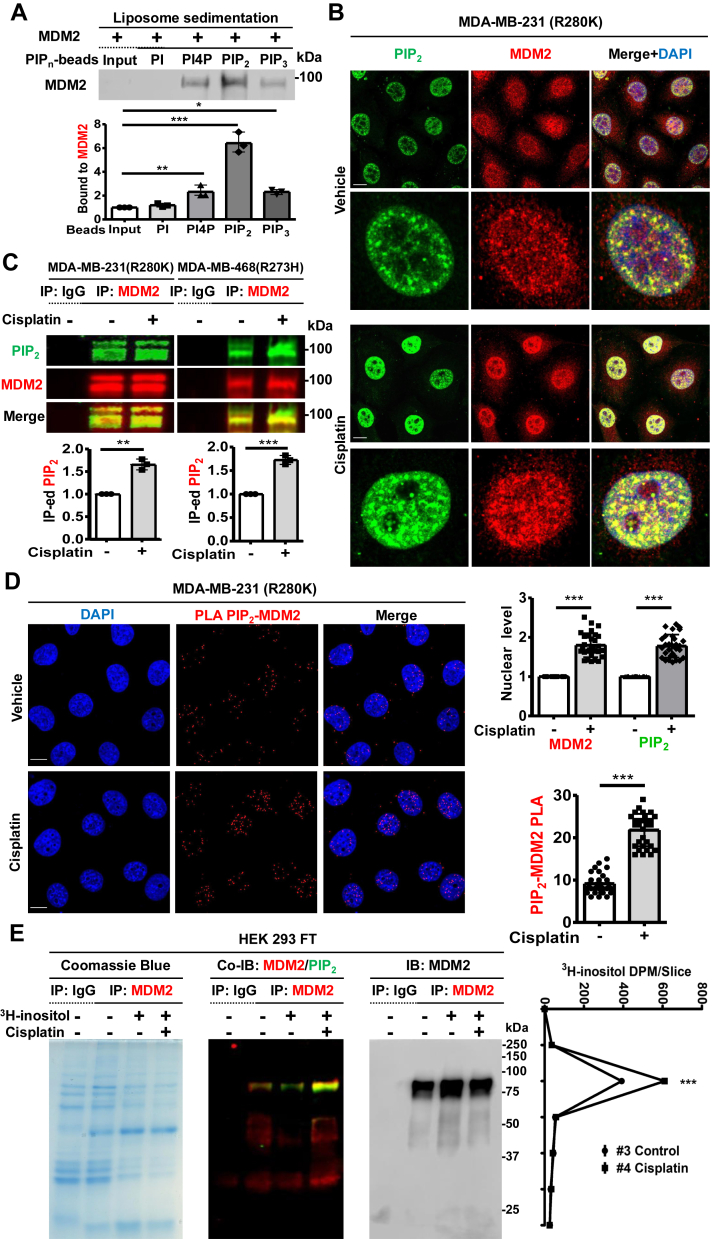
**PIP_2_ associates with MDM2 in the nucleus in response to stress.***A*, MDM2 recombinant protein (0.1 μg) was incubated with 1.0 nM liposomes containing the indicated phosphoinositides. Liposomes were sedimented, and the associated MDM2 was analyzed by immunoblotting (IB). The MDM2 IB intensity was quantified, and the graph is shown as mean ± standard deviation(s.d.) of *n* = 4 independent experiments. *B*, confocal images of immunofluorescence (IF) staining against PIP_2_ and MDM2 in MDA-MB-231 cells treated with vehicle or 30 μM cisplatin (Cis) for 24 h. MDM2 and PIP_2_ antibodies were used for the immunostaining study: scale bars, 5 μm. *C*, MDA-MB-231, and MDA-MB-468 cells were treated with 30 μM cisplatin or vehicle for 24 h and then processed for immunoprecipitation (IP) of MDM2 and fluorescence IB. Fluorescence IP−IB detects stress-induced PIP_2_ association with endogenous MDM2. The PIP_2_ IB intensity was quantified, and the graph is shown as mean ± s.d. of *n* = 3 independent experiments. *D*, proximity ligation assay (PLA) of MDM2-PIP_2_ in MDA-MB-231 cells treated with vehicle or 30 μM cisplatin for 24 h. The nuclear PLA foci of MDM2-PIP_2_ were quantified. *n* = 30 cells pooled from 3 independent experiments, 10 cells per experiment. *E*, HEK293FT cells transiently transfected with MDM2 were treated with vehicle or 25 μCi/ml ^3^H-myo-inositol to metabolic labeling. Cells were treated with vehicle or 30 μM cisplatin for 24 h, then processed for Coomassie blue staining and IP of MDM2 and fluorescence IB. The gel was sliced by molecular weight corresponding to the adjacent graph and analyzed by liquid scintillation counting (LSC). *n* = 3 slices pooled from 3 independent experiments, 6 slices per experiment.

As MDM2 has clusters of positively charged amino acids consistent with one or more putative phosphoinositide interaction motifs (2, 18, 19), we utilized a liposome sedimentation assay to investigate MDM2 binding to multiple phosphatidylinositol phosphate (PIP_n_) isomers. Recombinant MDM2 protein was incubated with PIP_n_-beads and MDM2-PIP_n_ liposomes, which were sedimented and analyzed by immunoblotting (IB) for MDM2. Although phosphatidylinositol (PI) did not bind MDM2, PI4P, PIP_2,_ and PIP_3_ bound to MDM2, with PIP_2_ exhibiting the most robust interaction (Fig. 1*A*). The specificity of the MDM2 antibody used throughout this study was validated by MDM2 KD in multiple cell lines (Fig. S1*A*), consistent with prior reports (20). Microscale thermophoresis (MST) confirmed direct binding of PIP_2_ to MDM2 (K_d_ 352 ± 3 nM, Table 1*A*). Consistent with their binding *in vitro*, cisplatin treatment increased nuclear levels of MDM2 and PIP_2_ and their nuclear co-localization as determined by immunofluorescence (IF) staining (Fig. 1*B*). Additionally, genotoxic stress increased the amount of PIP_2_ that co-immunoprecipitated with MDM2 across a panel of mutant p53 or p53-null cancer cell lines, while this interaction was decreased by cisplatin in a p53 wild-type cell line (Figs. 1*C* and S1*B*). Proximity ligation assay (PLA), which detects two closely located epitopes within 40 nm, revealed that the majority of the MDM2-PIP_2_ and p53-MDM2 foci were localized in the nucleus, with both signals increasing in response to cisplatin (Figs. 1*D* and S1*C*). Furthermore, we confirmed the stable association of MDM2 and PIP_2_ by metabolic labeling cells with [^3^H]*my*o-inositol, which resulted in the incorporation of [^3^H]*my*o-inositol into MDM2. Liquid scintillation counting (LSC) demonstrated that [^3^H]*my*o-inositol was primarily detected in 75∼100 kDa gel slices, corresponding to the molecular weight of MDM2, in the [^3^H]*my*o-inositol-treated group (Fig. 1*E*). Notably, this incorporation of [^3^H]*my*o-inositol into MDM2 was not disrupted by harsh denaturing conditions and was enhanced by cisplatin treatment. Collectively, these data indicate that PIP_2_ directly binds to MDM2 with high affinity *in vitro* and stably associates with MDM2 in the nucleus of cells in response to stress.

**Table 1:** MST Analysis of the binding affinity of MDM2 to PIPKIα, αBC, HSP27, PIP_2_ and p53

A	
Binding partners	Binding affinity with MDM2 (K_d_)
PIPKIα	28 ± 3 nM
αBC	58 ± 5 nM
HSP27	50 ± 7 nM
PIP_2_	352 ± 3 nM
p53	8 ± 1 nM


B		
Binding partners	Binding affinity with	
	αBC (K_d_)	HSP27 (K_d_)
His-MDM2	58 ± 5 nM	50 ± 7 nM
FLAG-MDM2	7 ± 1 nM	126 ± 4 nM
His-p53	66 ± 5 nM	127 ± 4 nM
FLAG-p53	14 ± 1 nM	38 ± 3 nM

*A*, Interaction of fluorescence-labeled MDM2 and non-labeled PIPKIα, αBC, HSP27, PIP_2,_ and p53 was evaluated using microscale thermophoresis (MST). MST traces of a constant concentration of fluorescence-labeled MDM2 (target, 5 nM) incubated with increasing concentrations to 500 nM of PIPKIα, αBC, HSP27, PIP_2,_ and p53 (ligand) were measured and used for calculating the binding affinity. MST revealed direct binding of MDM2 to PIPKIα, αBC, HSP27, PIP_2_, and p53 at the indicated K_d_ values. MST was performed using a Monolith NT.115 Pico, and the binding affinity was auto-generated using Control v1.6 software. n = 3 independent experiments.

*B*, Direct binding of fluorescence-labeled αBC and HSP27 (target, 5 nM) with increasing amounts to 500 nM of nonlabeled His-MDM2, FLAG-MDM2, His-p53, and FLAG-p53 (ligand) was analyzed using MST as in *A*.

### PIPKIα interacts with MDM2 and regulates PIP_2_ association

**Figure 2:**
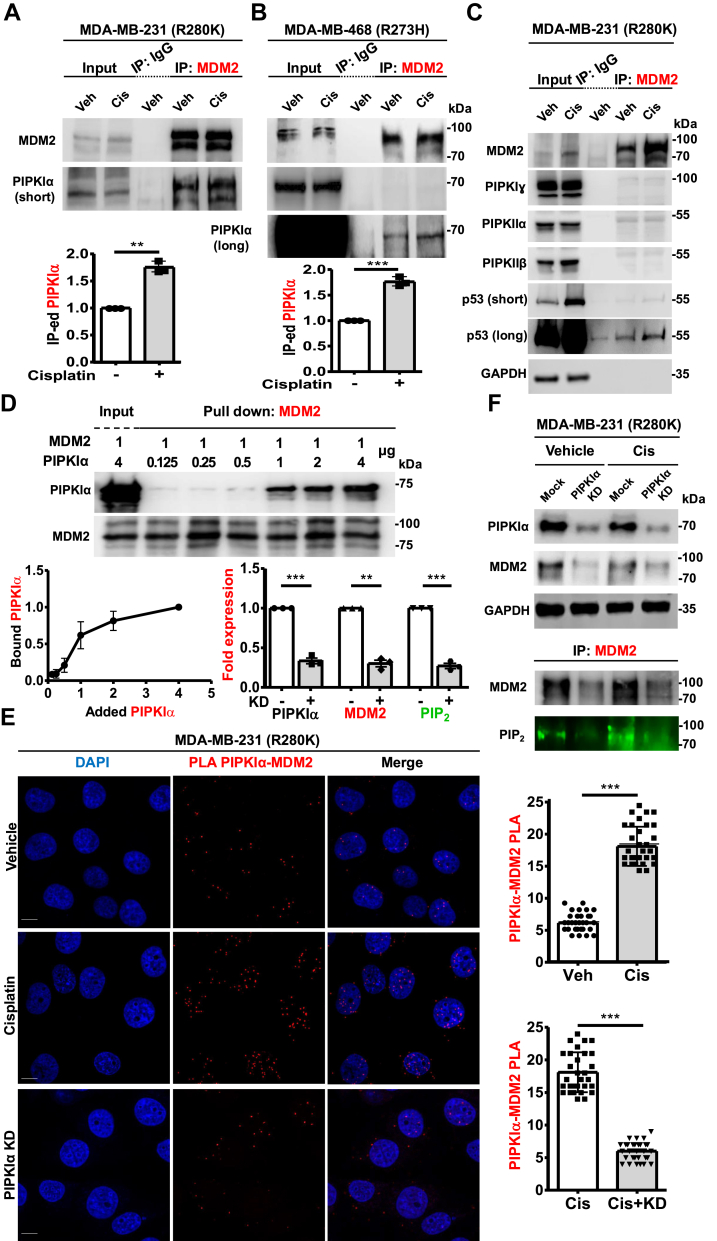
**PIPKIα interacts with MDM2 and regulates PIP_2_ association.***A* and *B*, Co-IP of MDM2 with PIPKIα from MDA-MB-231 (*A*) and MDA-MB-468 cells (*B*) treated with 30 μM cisplatin or vehicle for 24 h. Data shown represent three independent experiments. The PIPKIα IB intensity was quantified, and the graph is shown as mean ± s.d. of n = 3 independent experiments. Veh, vehicle; Cis, cisplatin-treated; short, short-time exposure; long, long-time exposure. *C*, Co-IP of endogenous MDM2 from MDA-MB-231 cells treated with vehicle or 30 μM cisplatin for 24 h. The MDM2, PIPKIα, PIPKIγ, PIPKIIα, PIPKIIβ, p53, and GAPDH IPed by MDM2 were analyzed by IB. short, short-time exposure; long, long-time exposure. *D*, recombinant MDM2 protein (1 μg) was incubated with 0.125, 0.25, 0.5, 1, 2 or 4 μg of PIPKIα protein. MDM2 was pulled down, and the binding with PIPKIα was analyzed with an anti-PIPKIα antibody. The graphs are shown as mean ± s.d. of n = 3 independent experiments. *E*, Quantification of nuclear PLA of PIPKIα-MDM2 in MDA-MB-231 cells treated with vehicle, cisplatin, or cisplatin plus siRNAs targeting PIPKIα for KD. For cisplatin treatment, 30 μM cisplatin was added for 24 h. PIPKIα KD was achieved using siRNAs for 24 h, followed by 24 h cisplatin treatment. n = 30 cells pooled from 3 independent experiments, 10 cells per experiment. Veh, vehicle; Cis, cisplatin-treated, KD; PIPKIα KD. *F*, MDA-MB-231 cells were transfected with siRNAs targeting PIPKIα. After 24 h of transfection, cells were treated with 30 μM cisplatin for 24 h. IB analyzed the expression of the indicated proteins, IBs were quantified, and the graph is shown as mean ± s.d. of n = 3 independent experiments. Cis, cisplatin-treated; KD, knockdown; Mock, empty vector.

As PIPKIα binds p53 and is required to generate PIP_2_ coupled to p53 (2, 3, 12), we examined its potential functional role in regulating the formation of MDM2-PIP_2_ complexes. Consistent with this idea, PIPKIα co-IPed with MDM2 in a panel of wild-type p53, mutant p53, and p53-null cancer cells (Figs. 2*A*, *B* and S2). In contrast, PIPKIγ, PIPKIIα, and PIPKIIβ, which also contribute to the nuclear PIP_2_ pool (21), interacted minimally or not at all with MDM2 (Figs. 2*C* and S2).

To elucidate whether PIPKIα binds directly to MDM2, a fixed concentration of recombinant MDM2 was incubated with increasing amounts of recombinant PIPKIα and pulled down with anti-MDM2 agarose. IB revealed saturable binding between PIPKIα and MDM2 (Fig. 2*D*). MST confirmed a direct high-affinity interaction between PIPKIα and MDM2 (K_d_ 28 ± 3 nM, Table 1*A*). To investigate this interaction in cells, siRNA-mediated knockdown (KD) of PIPKIα was performed as previously described (3, 12). PLA demonstrated that PIPKIα associated with MDM2 predominantly in the nucleus and that these complexes were increased by genotoxic stress and decreased by PIPKIα KD (Fig. 2*E*). Notably, PIPKIα KD also reduced MDM2 levels and PIP_2_ association with MDM2 under basal conditions and in response to stress (Figs. 2*F* and S3*A* and *B*). These results indicate that PIPKIα binds MDM2 in the nucleus and is required to generate nuclear MDM2-PIP_2_ complexes.

### MDM2 associates with sHSPs in the nucleus

**Figure 3:**
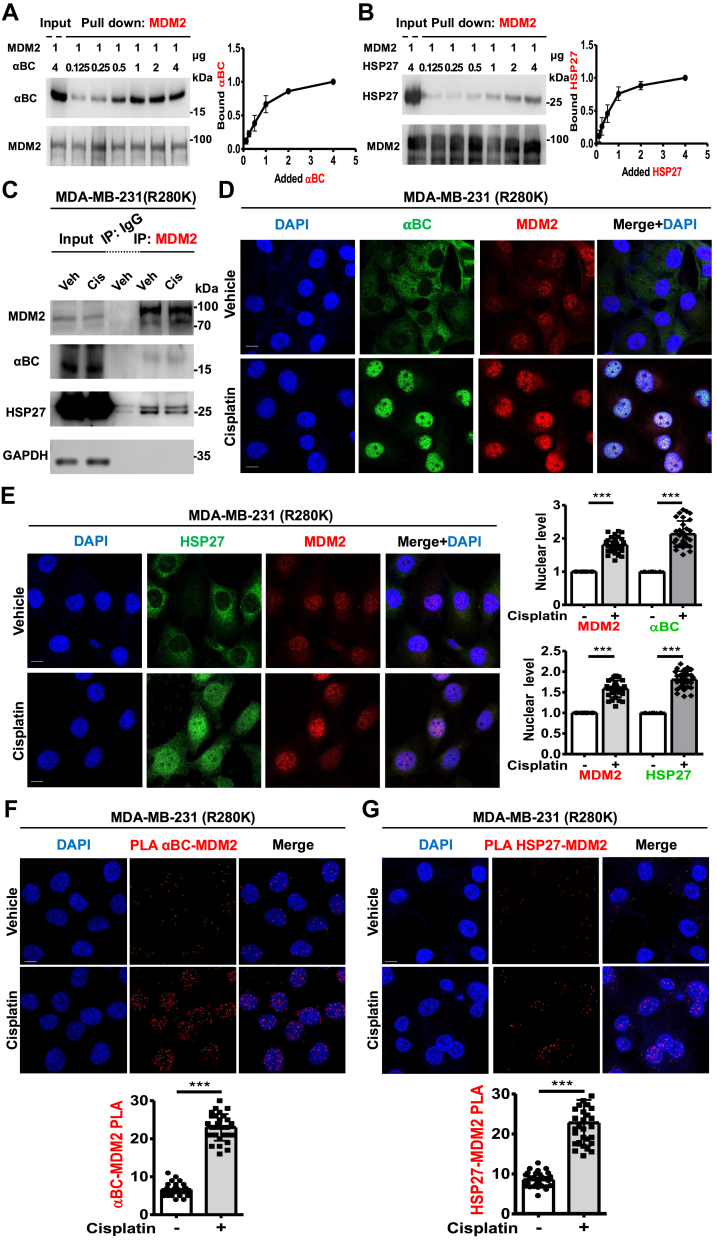
**MDM2 associates with sHSPs in the nucleus.***A* and *B*, recombinant MDM2 protein (1 μg) was incubated with 0.125, 0.25, 0.5, 1, 2, or 4 μg of αBC (*A*) and HSP27 (*B*) proteins. MDM2 was pulled down, and the association with αBC and HSP27 was analyzed using anti-αBC and anti-HSP27 antibodies. The graphs are shown as mean ± s.d. of n = 3 independent experiments. *C*, Co-IP of endogenous MDM2 from MDA-MB-231 cells treated with vehicle or 30 μM cisplatin for 24 h. IB analyzed the MDM2, αBC, HSP27, and GAPDH IPed by MDM2. Veh, vehicle; Cis, cisplatin-treated. *D* and *E*, Confocal images of IF staining against αBC (*D*) and HSP27 (*E*) along with MDM2 in MDA-MB-231 cells treated with vehicle or 30 μM cisplatin for 24 h. The nuclear levels of MDM2, αBC, and HSP27 were normalized to vehicle-treated cells, and their nuclear co-localization was determined by immunofluorescence. n = 30 cells pooled from 3 independent experiments, 10 cells per experiment. *F* and *G*, PLA of αBC–MDM2 (*F*) and HSP27-MDM2 (*G*) in MDA-MB-231 cells treated with vehicle or 30 μM cisplatin for 24 h. The nuclear PLA foci of αBC–MDM2 and HSP27-MDM2 were quantified. n = 30 cells pooled from 3 independent experiments, 10 cells per experiment.

The sHSPs, αBC and HSP27, bind p53 in the nucleus and promote the stability of p53 (3). To investigate whether sHSPs bind MDM2, a fixed concentration of recombinant MDM2 protein was incubated with increasing amounts of sHSPs, and the complex was then pulled down with anti-MDM2 agarose. IB showed saturable interactions between the sHSPs and MDM2 (Fig. 3, *A* and *B*). High-affinity interactions between MDM2 and αBC (K_d_ 58 ± 5 nM) and HSP27 (K_d_ 50 ± 7 nM) were confirmed by MST (Table 1*A*). Each sHSP Co-IPed with MDM2 in mutant p53, wild-type p53, or p53-null cancer cells (Figs. 3*C* and S2). IF staining revealed that αBC and HSP27 translocate to the nucleus in response to genotoxic stress, consistent with prior reports (22, 23, 24) and co-localize with MDM2 in the nucleus (Fig. 3, *D* and *E*). Stress increased the formation of MDM2-αBC and MDM2-HSP27 complexes in the nucleus as determined by PLA (Fig. 3, *F* and *G*). These data demonstrate that αBC and HSP27 directly bind MDM2 in the nucleus in response to genotoxic stress.

### PIP_2_ regulates the interaction of MDM2, sHSPs, and p53

**Figure 4:**
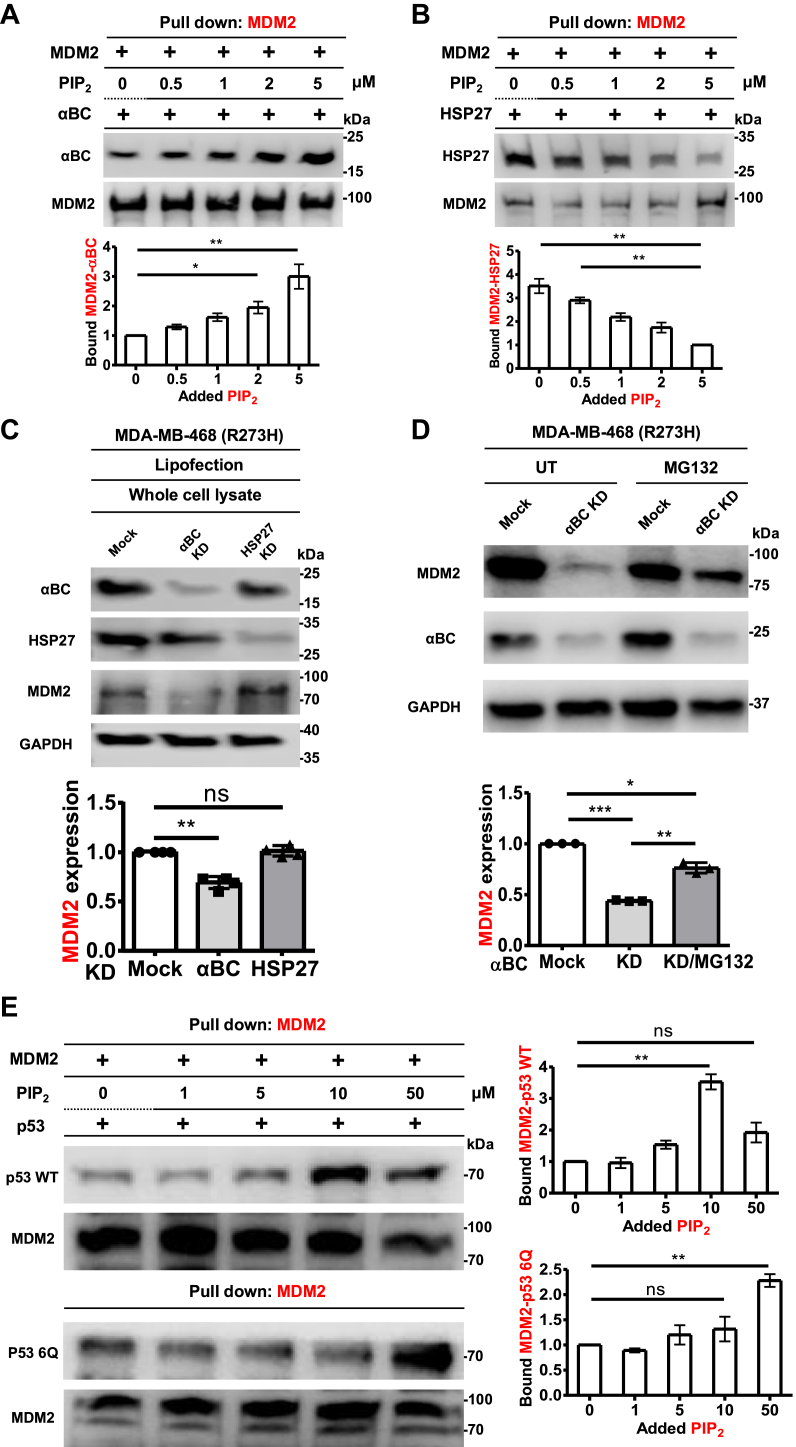
**PIP_2_ regulates the interaction of MDM2, sHSPs and p53 and controls MDM2 stability.***A* and *B*, 0.1 μg of MDM2 recombinant protein and 0.1 μg of αBC (*A*) or HSP27 (*B*) protein were incubated with 0, 0.5, 1, 2, or 5 μM PIP_2_. MDM2 was pulled down, and the associated αBC or HSP27 was analyzed with an anti-αBC or an anti-HSP27 antibody. The graphs are shown as mean ± s.d. of *n* = 3 independent experiments. *C*, MDA-MB-468 cells were transfected with siRNAs for αBC or HSP27 for 48 h. An empty vector (Mock) was used as a negative control. Expression of the indicated proteins was analyzed by IB MDM2 IBs were quantified, and the graph is shown as mean ± s.d. of n = 4 independent experiments. KD, knockdown. *D*, MDA-MB-468 cells were transfected with siRNAs for αBC for 48 h. Before harvesting, cells were treated with 10 μM of MG132 for 4 h. Empty vector (Mock) was used as a negative control. Expression of the indicated proteins was analyzed by IB, and MDM2 IBs were quantified. The graph is shown as mean ± s.d. of n = 3 independent experiments. *E*, recombinant MDM2 protein (0.1 μg) and GST-tagged p53 WT or PIP2-binding-defective p53 6Q mutant (p53 6Q, 0.1 μg) were incubated with 0, 1, 5, 10, or 50 μM PIP_2_. MDM2 was pulled down, and the associated p53 was analyzed with an anti-p53 antibody. The graphs are shown as mean ± s.d. of *n* = 3 independent experiments. p53 WT, wild type p53; p53 6Q, p53 6Q mutant.

To examine whether PIP_2_ regulates the association of sHSPs with MDM2 as previously demonstrated for sHSPs and p53 (3), increasing amounts of PIP_2_ were added to recombinant sHSPs and MDM2, and the complex was then pulled down with anti-MDM2 agarose. Intriguingly, while PIP_2_ enhanced the binding of both αBC and HSP27 to p53 (3), it differentially regulated sHSP binding to MDM2 (Fig. 4, *A* and *B*). Specifically, PIP_2_ increased αBC binding to MDM2 but decreased HSP27 binding to MDM2. These data suggest that αBC and HSP27 may have different roles in regulating MDM2. To investigate this possibility, αBC and HSP27 were each KDed, and MDM2 levels were assessed (Fig. 4*C*). The siRNAs used selectively targeted either αBC or HSP27 without off-target effects on the other sHSP. αBC KD modestly decreased MDM2 protein levels, whereas HSP27 KD had little effect on MDM2 levels (Figs. 4*C* and S3*C*). Since PIP_2_ enhances αBC binding to MDM2 and increases MDM2 protein levels, we postulated that αBC promotes MDM2 stability by inhibiting its degradation by the ubiquitin-proteasome pathway. Consistent with this idea, the proteasome inhibitor MG132 attenuated the effects of αBC KD on MDM2 levels (Fig. 4*D*). These data indicate that PIP_2_ differentially regulates sHSPs binding to MDM2 and that αBC regulates MDM2 stability by blocking its proteasome degradation.

Next, we explored the effects of PIP_2_ on the MDM2-p53 interaction using an *in vitro* pull-down assay. Recombinant GST-p53 protein and MDM2 were incubated with increasing concentrations of PIP_2,_ and the amount of p53 that co-IPed with MDM2 was determined (Fig. 4*E*). PIP_2_ enhanced the binding of MDM2 to p53, with maximal effects observed at 10 mM PIP_2_. Since both MDM2 and p53 associate with PIP_2_, we used a PIP_2_ binding-defective p53 6Q mutant (p53 6Q), which has five lysine residues and one arginine in the p53 CTD mutated to glutamine (3). Notably, the incubation of PIP_2_ with MDM2 and p53 6Q resulted in the enhanced binding of MDM2 to p53, indicating that PIP_2_ binding to MDM2 specifically increases its interaction with p53. Ectopically expressed PIP2-binding-defective p53 mutants (6Q and p53 R379Q mutant) in p53-null H1299 cells also bound to MDM2, consistent with prior results (Fig. S3*D*) (3). The findings indicate that PIP_2_ regulates the interaction between MDM2 and p53, at least in part, by binding to MDM2.

**Figure 5:**
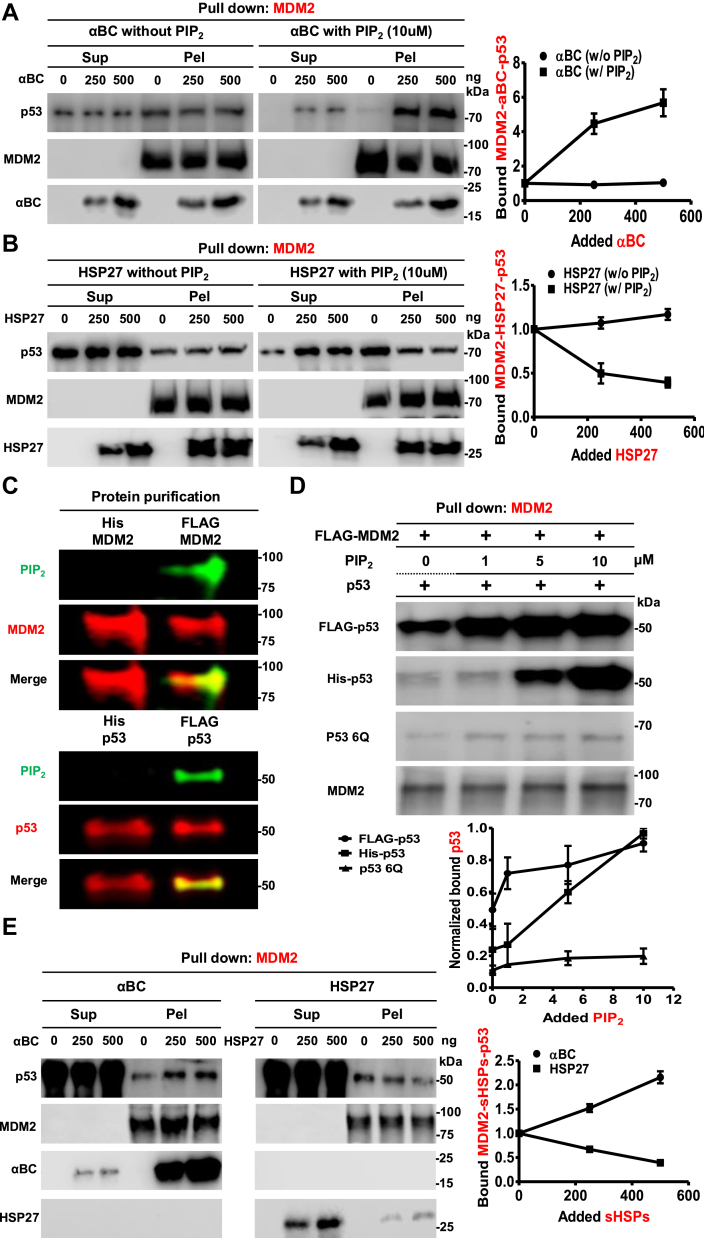
**PIP_2_ and sHSPs regulate the MDM2-p53 interaction.***A* and *B*, 0.1 μg of MDM2 and GST-p53 recombinant protein were incubated with gradually increasing amounts (0, 250, 500 ng) of αBC (*A*) or HSP27 (*B*) in the presence or absence of 10 μM of PIP_2_. After pulling down with MDM2 antibody-conjugated beads, the bound p53, αBC and HSP27 were analyzed with an anti-p53, anti-αBC or anti-HSP27 antibody in the supernatant and pellet. The graphs are shown as mean ± s.d. of *n* = 3 independent experiments. sup, supernatant; pel, pellet. *C*, 0.5 μg Bacteria (His-tagged) and mammalian-expressed (FLAG-tagged) MDM2 (*A*) and p53 (*B*) proteins were analyzed by fluorescence IB. Fluorescence IB detects purified proteins and PIP_2_ stably associated to proteins. The MDM2 and PIP_2_ association is shown in the merged image. *D*, recombinant MDM2 protein (0.1 μg) and FLAG-, His-p53, or GST-p53 6Q mutant proteins were incubated with 0, 1, 5, or 10 μM PIP_2_. MDM2 was pulled down, and the associated different p53 was analyzed with an anti-p53 antibody. The graphs are shown bound p53 normalized to the strongest signal (His-p53 with 10 μM PIP_2_) as mean ± s.d. of *n* = 3 independent experiments. p53 6Q, p53 6Q mutant. *E*, 0.1 μg of FLAG-MDM2 and p53 recombinant protein were incubated with gradually increasing amounts (0, 250, 500 ng) of αBC or HSP27. After pulling down with MDM2 beads, the bound p53, αBC and HSP27 were analyzed with an anti-p53, anti-αBC or anti-HSP27 antibody in the supernatant and pellet. The graphs are shown as mean ± s.d. of *n* = 3 independent experiments. FLAG-tagged proteins; mammalian-derived proteins.

To determine whether sHSPs contribute to regulating MDM2–p53 interaction by PIP_2_, we performed pull-down experiments using recombinant proteins in the presence and absence of PIP_2_. Without PIP_2_, both sHSPs had little effect on MDM2 binding to p53 (Fig. 5, *A* and *B*). However, in the presence of PIP_2_, αBC enhanced the interaction between MDM2 and p53, whereas HSP27 inhibited their association. These findings suggest that sHSPs differentially regulate MDM2–p53 binding through a PIP_2_-dependent mechanism.

One notable limitation of these experiments is that incubating PIP_2_ with bacteria-derived recombinant p53 and MDM2 in cell-free systems leads to binding, but PIP_n_ has rarely been found in bacteria; hence, bacteria are not a suitable model to demonstrate the stable association of PIP_2_ to these proteins observed in eukaryotic cellular systems (25). Therefore, we purified MDM2 and p53 from mammalian HEK293FT cells to address this issue. PIP_2_ was stably associated with FLAG-tagged MDM2 and p53 produced in mammalian cells, but not His-tagged MDM2 and p53 produced in *Escherichia coli* (Fig. 5*C*). As such, these recombinant proteins produced in mammalian cells enable investigation into the nature of the PIP_2_ linkage. To understand how PIP_2_ modulates the interaction between bacteria- and mammalian-derived MDM2 and p53, we performed an *in vitro* binding assay using FLAG-MDM2 and various forms of p53, with gradually increasing concentrations of PIP_2_. Intriguingly, in the absence of PIP_2_, FLAG-MDM2 showed robust binding only to FLAG-p53, which gradually increased with addition of PIP_2_. In contrast, His-p53 exhibited minimal binding to FLAG-p53 in the absence of PIP_2_ but showed a dramatic increase in binding upon PIP_2_ addition. However, the PIP-binding-defective p53 6Q mutant displayed minimal binding and no significant change was detected by adding PIP_2_ (Fig. 5*D*). These results suggest that PIP_2_ linkage and/or PIP_2_ binding promotes the interaction between MDM2 and p53.

Additionally, we analyzed the binding affinities between sHSPs and FLAG-tagged proteins. Consistent with the *in vitro* binding data (Fig. 4*A*), FLAG-MDM2 exhibited an increased binding affinity for αBC (K_d_ = 7 ± 1 nM) and a decreased affinity for HSP27 (K_d_ = 125 ± 9 nM) compared to His-MDM2 as determined by MST (Table 1*B*). In contrast, FLAG-p53 demonstrated an increased binding affinity for both αBC (K_d_ = 14 ± 1 nM) and HSP27 (K_d_ = 38 ± 3 nM). An *in vitro* binding assay involving FLAG-MDM2, FLAG-p53, and sHSPs revealed that the amount of p53 pulled down with MDM2 varied, yet the pattern remained consistent with previous observation: αBC enhanced the interaction between FLAG-MDM2 and p53, whereas HSP27 reduced their binding (Fig. 5*E*). Taken together, these data from both His- and FLAG-proteins indicate that PIP_n_-linked MDM2 mimics several features of PIP_n_ binding, including the differential recruitment of sHSPs and enhanced interaction with p53.

### PIP_2_ and sHSPs regulate the function of MDM2

**Figure 6:**
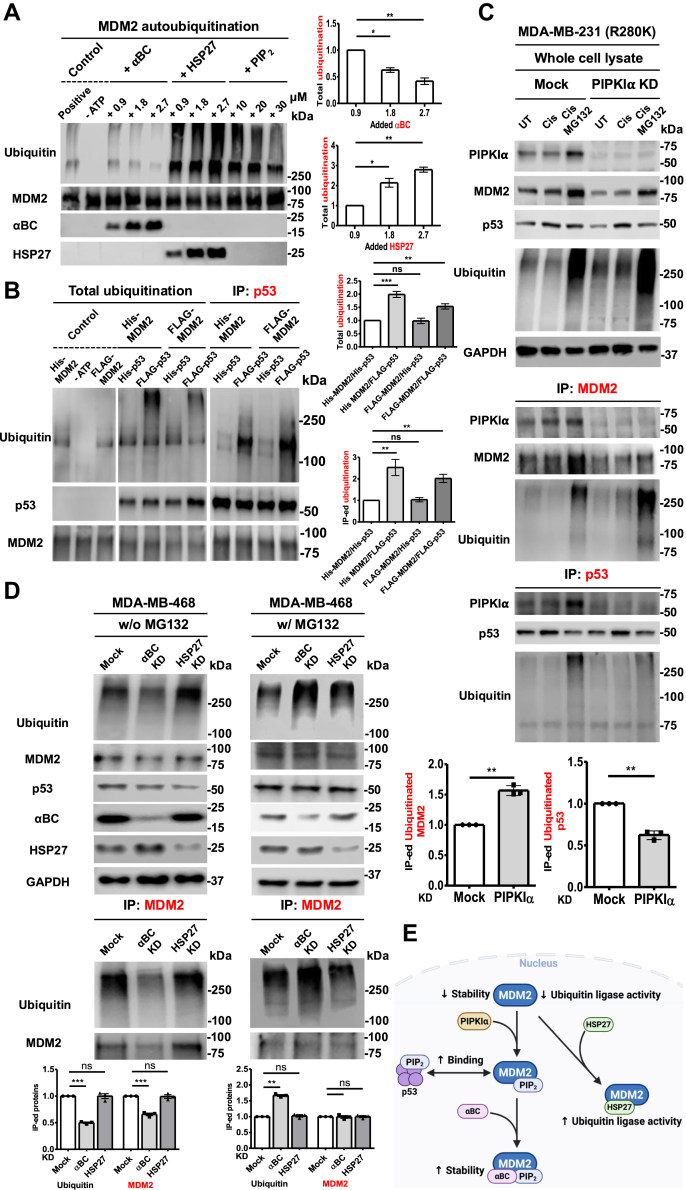
**PIP_2_ and sHSPs regulate the ubiquitination function of MDM2.***A*, for MDM2 autoubiquitination, 100 nM of E1 enzyme, 1 μM of E2 enzyme, 1 μM of MDM2, E3 ligase reaction buffer, 10 mM of MgATP solution and 100 μM of ubiquitin were incubated with different concentrations of αBC, HSP27 (0.9, 1.8, or 2.7 μM), or PIP_2_ (10, 20, or 30 μM) for 1 h. Ubiquitin, MDM2, αBC and HSP27 were analyzed by IB, and ubiquitin IBs were quantified. The graphs are shown as mean ± s.d. of *n* = 3 independent experiments. *B*, for *in vitro* ubiquitination, 100 nM of E1 enzyme, 1 μM of E2 enzyme, 1 μM of His- and FLAG-tagged MDM2, E3 ligase reaction buffer, 10 mM of MgATP solution and 100 μM of ubiquitin were incubated with 1 μM of His- and FLAG-tagged p53 for 1 h. For IP, samples were incubated with anti-p53-conjugated agarose overnight. Ubiquitin, MDM2, and p53 were analyzed by IB, and ubiquitin IBs were quantified. The graphs are shown as mean ± s.d. of *n* = 3 independent experiments. *C*, MDA-MB-231 cells were transfected with siRNAs for PIPKIα and treated with vehicle or 30 μM cisplatin for 24 h. Empty vector (Mock) was used as a negative control. After cells were treated with 10 μM of MG132 for 4 h, cells were harvested for IP of MDM2 and p53. Expression of the indicated proteins was analyzed by IB, and ubiquitin IBs were quantified. The graph is shown as mean ± s.d. of n = 3 independent experiments. KD, knockdown; UT, untreated; Cis, cisplatin treated; Cis/MG132, Cisplatin/MG132 treated. *D*, MDA-MB-468 cells were transfected with siRNAs for αBC and HSP27 for 48 h in absence or presence of MG132. Empty vector (Mock) was used as a negative control. Cells were processed for IP of MDM2. IB was used to analyze indicated proteins, and ubiquitin IBs were quantified. The graphs are shown as mean ± s.d. of n = 3 independent experiments. KD, knockdown. *E*, a model of PIP_2_ regulation of the interaction between MDM2 and sHSPs, which differentially control the stability of MDM2 and function of of MDM2. In the presence of PIPKIα, PIP_2_ is linked to MDM2, which recruits αBC to MDM2 to stabilize it and enhances p53 binding. Conversely, in the absence of PIPKIα and PIP_2_, HSP27 is recruited to MDM2, which increases the ubiquitin E3 ligase activity of MDM2.

We next examined whether sHSPs regulated the ubiquitination of MDM2. A unique characteristic of RING finger E3 ubiquitin ligases, including MDM2, is that they regulate the ubiquitination of their targets and themselves (autoubiquitination) (26). As such, we utilized an *in vitro* ubiquitination assay to determine the effects of PIP_2_ and sHSPs on MDM2 autoubiquitination. Compared to the positive control, MDM2 *in vitro* ubiquitination was decreased by αBC but increased when HSP27 and PIP_2_ were added individually (Fig. 6*A*). Notably, sHSPs regulated MDM2 *in vitro* ubiquitination in a dose-dependent manner, while the effects of PIP_2_ were dose-independent. Moreover, adding PIP_2_ to this cell-free MDM2 autoubiquitination assay did not alter the impact of either αBC or HSP27 on MDM2 autoubiquitination (Fig. S4*A*). Similar findings were observed for total *in vitro* ubiquitination when both p53 and MDM2 with sHSPs or PIP_2_ were added (Fig. S4*B*). To investigate the effect of PIP_2_ linkage on ubiquitination, MDM2 and p53 proteins expressed in mammalian cells were used in an *in vitro* ubiquitination assay and compared with *E. coli-expressed* proteins. PIP_2_ linkage had minimal impact on ubiquitination activity of mammalian-derived MDM2, while it significantly increased the total ubiquitination and p53 ubiquitination (Fig. 6*B*). These data demonstrate that both binding and linkage of PIP_2_ and sHSPs regulate MDM2 and p53 ubiquitination, with αBC inhibiting ubiquitination and HSP27 and PIP_2_ enhancing ubiquitination of MDM2 and/or p53.

Next, we investigated the regulation of MDM2 ubiquitination in cellular models. MDM2 was IPed after PIPKIα KD in the presence or absence of Cisplatin and MG132. IB showed the successful KD of PIPKIα, increased total and MDM2 ubiquitination, and reduced p53 ubiquitination (Fig. 6*C*). Along with the increased accumulation of ubiquitinated MDM2, MDM2 expression was decreased and restored by MG132 treatment. Similar results were observed in A549 cells expressing wild-type p53 (Fig. S4*C*). These data indicate that KD of PIPKIα enhances MDM2 ubiquitination, likely promoting degradation of MDM2 *via* the ubiquitin-proteasome pathway, which in turn leads to decreased ubiquitinated p53. Consistent with prior reports (3), PIPKIα KD reduced p53 levels, which may reflect reduced sHSP recruitment/stabilization.

To identify the functional role of sHSPs in the ubiquitination of MDM2, we transfected siRNAs targeting sHSPs in p53 mutant MDA-MB-468 cells to measure degradation of ubiquitinated MDM2 in the absence of MG132 and accumulation of ubiquitinated MDM2 in the presence of MG132 (Fig. 6*D*). In the absence of MG132, αBC KD resulted in decreased accumulation of ubiquitinated MDM2 and decreased level of MDM2 due to degradation. Consistent with previous reports (3), p53 expression decreased following sHSPs KD. However, following MG132 treatment, αBC KD led to an increased accumulation of ubiquitinated MDM2, and MDM2 levels were similar to that in the control group. Despite MG132 treatment, HSP27 KD did not significantly alter MDM2 ubiquitination and/or MDM2 levels. MG132 treatment restored p53 levels, likely by inhibiting proteasome-mediated degradation. These data indicate that sHSPs have differential effects on MDM2 ubiquitination *in vitro* and in cellular models.

Previous studies have shown that MDM2 regulates several additional targets by ubiquitination, including FOXO3a and histone H2A/H2B, in a p53-independent manner (27, 28). To test whether PIP_2_ association *via* PIPKIα modulated targets MDM2 targets in a p53-independent system, we employed PIPKIα KD using siRNA in the p53-null H1299 cell line (Fig. S4*D*). Consistent with our earlier findings, PIPKIα KD led to a reduction of MDM2 levels, stabilizing its targets, FOXO3a and H2B. These data suggest that the association of PIP_2_ with MDM2 also likely regulates the ubiquitination of other MDM2 substrates, potentially expanding the functional effects of the MDM2-PIP_2_ complex.

## Discussion

Since the discovery of PIP_n_s in the nucleus (29), their function in the nucleus has been intensively investigated (5, 12, 19, 30, 31, 32, 33, 34, 35). Recently, the lipid kinase PIPKIα and its product PIP_2_ were reported to bind the CTD of stress-activated wild-type p53 and mutant p53 and stabilize p53 by recruiting the sHSPs αBC and HSP27 to the p53-PIP_2_ complex (3). Since the stability of wild-type p53 is regulated by E3 ubiquitin ligase MDM2 (36), we began investigating its potential functional role in regulating the effects of PIP_2_ on p53 stability. We discovered that multiple PIP_n_s interact with MDM2. Specifically, PIP_2_, and to a lesser extent PI4P, and PIP_3_, bind to MDM2 in response to stress. The association of PIP_2_ with MDM2 requires PIPKIα, which binds directly to MDM2 with high affinity (K_d_ 28 ± 3 nM) to regulate the formation of MDM2-PIP_2_ complexes, a mechanism highly reminiscent of their role in regulating p53 stability (3). The association between MDM2 and PIP_2_ is very stable as it can be detected by IB and withstands harsh denaturation with reducing agents, high temperature, and SDS-polyacrylamide gel electrophoresis, similar to other PIP_2_ effectors (3, 37, 38). Moreover, metabolic labeling of cells with [H^3^]*myo*-inositol, which is metabolized solely to phosphoinositides, results in the stable incorporation of [H^3^] label into MDM2 in response to stress, and this linked [H^3^]PIP_n_ is also resistant to harsh denaturing conditions. These results indicate that PIP_2_ and other PIP_n_s stably associate with MDM2, likely by a covalent bond/novel posttranslational modification (PTM). The chemical nature and location of these putative PTMs on MDM2 and p53 are currently being investigated by multiple orthogonal approaches.

As previously reported, genotoxic stress induces the nuclear translocation of sHSPs (3, 22, 24, 39, 40), facilitating their interaction with nuclear MDM2. Although MDM2 interacts directly with both αBC and HSP27, PIP_2_ binding to MDM2 regulates its association with sHSPs. In contrast to p53, where PIP_2_ binding enhances the recruitment of αBC and HSP27, which both stabilize p53 (3), the PIP_2_ bound to MDM2 differentially regulates αBC and HSP27 recruitment, acting like a switch (Fig. 6*E*). Specifically, PIP_2_ binding to MDM2 enhances αBC binding, which stabilizes MDM2. In contrast, the association of PIP_2_ with MDM2 decreases HSP27 binding, which does not affect MDM2 stability. Moreover, recombinant αBC inhibits MDM2 autoubiquitination *in vitro*, while HSP27 enhances MDM2 autoubiquitination. To add to the complexity of these interactions, αBC and HSP27 also have divergent effects on the MDM2-p53 interaction, with αBC increasing the association of MDM2 with p53 by a PIP_2_-dependent mechanism and HSP27 having the opposite effect. These opposing effects of αBC and HSP27 enable exquisite fine-tuning of the regulation of the MDM2-p53 nexus by PIP_2_.

Since adding PIP_2_ to the *in vitro* binding assay incubation does not fully recapitulate the stable association of PIP_2_ to p53 and MDM2 observed in cells, we purified MDM2 protein produced in mammalian cells, which is PIP_n_-linked in contrast to bacterial-expressed MDM2 protein. Notably, PIP_n_-linked MDM2 mimicked many of the effects of PIP_n_ binding to MDM2. Specifically, mammalian-expressed MDM2 exhibited increased αBC interaction and diminished HSP27 association compared with the bacterial-expressed MDM2. Moreover, αBC enhanced the interaction between PIP_n_-linked MDM2 and p53, while HSP27 inhibited their interaction, recapitulating the effects of PIP_2_ and sHSP binding on the MDM2-p53 interaction. Strikingly, PIP_2_ linkage to MDM2 and p53 facilitated their interaction in the absence of PIP_2_, mimicking the effects of PIP_2_ binding, while the interaction between PIP_2_-linked MDM2 and p53 was further enhanced by adding PIP_2_. Collectively, these results underscore that the PIP_n_-linked MDM2 and p53 proteins from mammalian cells recapitulate the effects of PIP_2_ binding on the MDM2-p53 nexus.

Since the interaction between MDM2 and p53 is critical for p53 stability and oncogenic activity (41, 42, 43), our results unveil a new therapeutic target: the MDM2-PIP_2_-sHSPs complex, which controls the MDM2-p53 interaction. Importantly, PIP_n_ linkage or PIP_2_ binding selectively regulates sHSP interactions with MDM2 and p53 to control the stability of MDM2. Although studies have investigated how sHSPs promote the ubiquitination of diverse targets (44, 45, 46, 47, 48), no reports have demonstrated that sHSPs regulate MDM2 ubiquitination. Our data point to a model that nuclear PIP_n_ linkage/PIP_2_ acts as a highly tunable on-off switch that confers specificity to determine whether αBC or HSP27 is recruited to MDM2, resulting in opposing effects (Fig. 6*E*). αBC promotes MDM2 stability, inhibits its autoubiquitination activity and promotes the association of MDM2 and p53, while HSP27 promotes MDM2 autoubiquitination and inhibits the MDM2-p53 interaction. In addition, our results provide additional support for a novel PIP_n_ signaling pathway on proteins such as p53 (3, 12), the nuclear poly(A) polymerase Star-PAP (5, 49), NRF2 (50) and MDM2, where they function as ‘third messengers’ tightly linked to effector proteins to regulate their stability and function.

## Experimental procedures

### Cell culture and constructs

MDA-MB-231, MDA-MB-468, BT-20, A549, HCT116, H1299, and HEK293FT cells were purchased from ATCC (American Type Culture Collection). BT-20 cells were maintained in EMEM (#30-2003, ATCC) supplemented with 10% fetal bovine serum (#SH30910.03, Hyclone) and 1% penicillin/streptomycin (#15140-122, Gibco). All other cells were maintained in DMEM (#11995-065, Gibco) with the abovementioned supplements. All cell lines were routinely checked for *mycoplasma* contamination using the MycoAlert *Mycoplasma* Detection Kit (#LT07-318, Lonza), and only mycoplasma-negative cells were used. None of the cell lines is listed in the database of commonly misidentified cell lines maintained by the International Cell Line Authentication Committee (ICLAC). The FLAG-tagged wild-type p53, p53 6Q mutant, p53 R175H mutant, αBC, and HSP27 constructs were described previously (3). The MDM2 cDNA was purchased from OriGene. Constructs were transfected into mammalian cells using a lipid-based delivery system, Lipofectamine3000 (#L3000015, Thermo Fisher Scientific), according to the manufacturer’s instructions. Typically, 2 to 5 μg DNA and 6 to 10 μl lipid were used for transfections in 6-well plates. Transfection efficiency was determined by IB. For recombinant protein production, the His-tagged constructs for MDM2, αBC, HSP27, GST-tagged p53 6Q mutant, and PIPKIα were purchased from Genscript, and the recombinant proteins were purified as previously described (3). Recombinant GST-tagged p53 (#14-865) was purchased from MilliporeSigma.

### Antibodies and reagents

Monoclonal antibodies against PIP_2_ (clone 2C11, #Z-P045, Echelon Biosciences), PIP_2_ (clone KT10, #MSBS2283, MilliporeSigma), p53 (clone DO-1, #SC-126, Santa Cruz Biotechnology), HSP27 (clone F-4, #SC-13132, Santa Cruz Biotechnology), αBC (D6S9E, #45844, Cell Signaling), PIPKIIα (PIP4K2A, clone D83C1, #5527, Cell Signaling), GAPDH (clone 0411, #SC-47724, Santa Cruz Biotechnology), and polyclonal antibodies against MDM2 (clone D1V2Z, #86934, Cell Signaling), MDM2 (#AF1244, R&D Systems), αBC (#ab13497, Abcam), PIPKIα (PIP5K1A, #9693, Cell Signaling), PIPKIγ (PIP5K1C, #3296, Cell Signaling), PIPKIIβ (PIP4K2B, #9694, Cell Signaling), FOXO3a (clone 75D8, #2497, Cell Signaling), Histone H2A (#2578, Cell Signaling), Histone H2B (clone D2H6, #12364, Cell Signaling), Ubiquitin (P37, #58395, Cell Signaling) were utilized in this study. For conventional immunostaining and PLA analysis of phosphoinositides, anti-PIP_2_ (#Z-P045) antibodies were used. For immunostaining analyses and proximity ligation assay (PLA), antibodies were diluted at a 1:100 ratio. The ON-TARGETplus siRNA SMARTpool with 4 siRNAs in combination against human MDM2, αBC, HSP27, and PIPKIα were purchased from Dharmacon. As a control, a non-targeting siRNA was purchased from Dharmacon. RNAiMAX reagent (#13778150, Thermo Fisher Scientific) was utilized to deliver siRNAs into the cells, and knockdown efficiency was determined by IB. Cisplatin (#S1166) was purchased from Sellekchem.

### Immunoprecipitation and immunoblotting

Cells were washed once with ice-cold PBS (#14190-144, Gibco), and lysed in ice-cold RIPA lysis buffer system (#sc-24948, Santa Cruz Biotechnology) supplemented with 1 mM Na_3_VO_4_, 5 mM NaF, and 1x protease inhibitor cocktail (#11836153001, Roche). The cell lysates were sonicated at 16% amplitude for 5 to 10 s. After sonication, the cell lysates were incubated at 4 °C with continuous rotation for 30 min for lysis. Subsequently, lysates were centrifuged at maximum speed for 15 min to collect the supernatant. The protein concentration was measured by the Bradford protein assay (#5000201, BIO-RAD) and equal amounts of protein were loaded in each lane. For IB analyses, antibodies were diluted at a 1:1000 ratio except for p53 (clone DO-1, 1:5000) and GAPDH (clone 0411, 1:5000). For immunoprecipitation, cell lysates were incubated with 20 μl anti-MDM2 (SMP14, #SC-965 AC, Santa Cruz Biotechnology) mouse monoclonal IgG antibody-conjugated agarose at 4 °C for 24 h. Normal immunoglobulin (IgG)-conjugated agarose was used as a negative control (#sc-2343, Santa Cruz Biotechnology). After incubation, samples were washed three times with PBST (PBS with 0.05% Tween 20), and the precipitated protein complex was resuspended in SDS sample loading buffer. For IB, 5 to 20 μg of protein were loaded. Horseradish peroxidase (HRP)-conjugated antibodies were used for IB of immunoprecipitated complexes to avoid non-specific detection of immunoglobulin in the immunoprecipitated samples. HRP-conjugated p53 (#SC-126 HRP), HSP27 (clone F-4, #SC-13132 HRP), and GAPDH (clone 0411, #SC-47724 HRP) antibodies were purchased from Santa Cruz Biotechnology. Immunoblots were captured using the Odyssey Imaging System (LI-COR Biosciences), and the intensity of protein bands was quantified using ImageJ. The unsaturated exposure of immunoblot images was used for quantification with the appropriate loading controls as standards.

### Fluorescent IP-IB

Cells were lysed and immunoprecipitated as described above. The sample was then boiled at 95 °C for 10 min. For IB, 5 to 20 μg of protein were loaded. The protein complexes associated with MDM2 were resolved by SDS-PAGE and transferred onto a PVDF membrane (#IPVH00010, MilliporeSigma). The membrane was blocked with 3% BSA in PBS for 1 h at room temperature. For double fluorescent IP-IB detecting MDM2-PIP_2_ complex, anti-MDM2 rabbit monoclonal IgG antibody (clone D1V2Z, #86934, Cell Signaling) at 1:1000 dilution and anti-PIP_2_ mouse monoclonal IgM antibody (#Z-P045, Echelon Biosciences) at 1:1000 dilution were incubated together in blocking buffer and incubated with the membrane at 4 °C overnight. Then the membrane was washed three times with PBST for 10 min each time on the rocking incubator. For the secondary antibody incubation, goat anti-rabbit IgG antibody conjugated with IRDye 800CW fluorophore (#926-32211, LI-COR), detectable on the 800 nm wavelength channel of the Odyssey Fc Imaging System (LI-COR Biosciences), and goat anti-mouse IgM antibody conjugated with IRDye 680RD fluorophore (#926-68180, LI-COR), detectable on the 700 nm wavelength channel at 1:5000 dilution, were mixed together in blocking buffer and incubated with the membrane at room temperature for 2 h. The membrane was then washed three times with PBST for 10 min each time on the rocking incubator. The images were subsequently acquired using the 700 and 800 nm wavelength channels simultaneously on the Odyssey Fc Imaging System (LI-COR Biosciences). The MDM2-associated PIP_2_ complex was visualized by overlapping the 700 and 800 nm channels.

### Radio-detection of ^3^H-inositol metabolic labeling

Cells were seeded and maintained in an Opti-MEM (#31985070, Thermo Fisher Scientific) supplemented with 10% dialyzed FBS with a 10,000 molecular weight cut-off (#F0392, MilliporeSigma) and 1% Pen/strep (Cat#: 15140-122, Gibco). The cells were allowed to adhere overnight to reach 5 to 10% confluency the next day and were treated with 25 μCi/ml of *myo*-[2-^3^H]-inositol (#NET1156005MD, PerkinElmer Life Science). Normal myo-inositol was supplemented in the medium of the control group. When the cells reached 50% confluency, they were transfected with MDM2 constructs as described above for 24 h, followed by treatment with vehicle control or 30 μM Cisplatin. After 24 h treatment, the cells were processed for IP-IB as described above. Cell lysates were subjected to IB for Coomassie blue staining and IB as described previously. For Coomassie blue staining, after running SDS-polyacrylamide gel electrophoresis, gels were washed with distilled water and stained with Coomassie blue (#ab119211, Abcam) for 1 h. Subsequently, the gels were dissected into 1∼2 mm pieces for LSC. The dissected gels were dissolved in 500 μl of 30% hydrogen peroxide (#H1009, MilliporeSigma) at 55 °C overnight. After complete dissolution, the sliced gels were cooled down at room temperature. The dissolved samples were mixed with 10 ml of Hionic-Fluor (#6013319, PerkinElmer Life Science) in Glass Scintillation Vials (#50212992, Thermo Fisher Scientific). The samples were placed in a dark room for 30 min for dark adaptation and then were loaded into Tri-Crab Scintillation counter (#4910TR PerkinElmer Life Science). Disintegration per minute (DPM) was automatically generated by Perkin Elmer Quantasmart.

### *In vitro* binding assay

Recombinant proteins were expressed in BL-21(DE3) *E. coli* strain (#EC0114, Thermo Fisher Scientific). Cells were lysed by sonication in 1% Brij58. GST-tagged p53 6Q mutant was purified with GST Sepharose 4B (#17075604, Cytiva), while His-tagged MDM2, αBC, HSP27, and PIPKIα were purified with Ni-NTA-agarose (#166038887, Qiagen) as previously described (3). Recombinant FLAG-tagged p53 R248Q were expressed in HEK293FT cells and purified protein by Meng S. Choy and Wolfgang Peti, University of Connecticut, Farmington, CT, USA. FLAG-tagged MDM2 were expressed in HEK293FT cells. Cells were lysed, and proteins were purified using FLAG Purification Kit (CELLMM2-1KT, MilliporeSigma). After purification, eluates were dialysis to exchange buffer into PBS using a dialysis cassette (#66380, Thermo Fisher Scientific), flash-frozen, and stored at −80 °C. Recombinant GST-tagged p53 (#14-865) was purchased from MilliporeSigma. The synthetic PIP_2_ diC16 (#P-4516) was purchased from Echelon Biosciences. The binding assay was conducted in Tris-based MST buffer containing 50 mM Tris-HCl, pH 8.0, 50 mM NaCl, 80 mM KCl, and 0.05% Tween-20. Various recombinant proteins were incubated at different concentrations. For pull-down experiments, 20 μl anti-MDM2 antibody-conjugated agaroses (SMP14, #SC-965 AC, Santa Cruz Biotechnology) were used. Following overnight incubation at 4 °C, unbound proteins were removed by washing three times with MST buffer or collected for supernatant and pellet comparison. The protein complex was analyzed by IB. GraphPad Prism generated the quantitative graph. The images were processed using ImageJ.

### Liposome sedimentation assay

For the sedimentation assay, control (P-B000), PI-coated (P-B001), PI4P-coated (P-B004a), PIP_2_-coated (P-B045a), and PIP_3_-coated (P-B345a) beads were purchased from Echelon Biosciences. Recombinant MDM2 was purified as described above. The sedimentation assay was performed in Tris-based MST buffer containing 50 mM Tris-HCl, pH 8.0, 50 mM NaCl, 80 mM KCl, and 0.05% Tween-20 by incubating 0.1 μg MDM2 recombinant protein with 10 μl lipid-coated beads. After overnight incubation at 4 °C, unbound MDM2 was removed by washing three times with MST buffer, and the sedimentations were resuspended with an equal amount of SDS sample buffer as supernatant. Subsequently, samples were resolved by SDS–PAGE and proteins associated with the liposomes were detected by IB.

### Microscale thermophoresis (MST) assay

The MST assay was utilized to measure the binding affinity of purified recombinant proteins *in vitro, as described previously* (12). The target protein was fluorescently labeled using the Monolith Protein Labeling Kit RED-NHS second Generation (#MO-L011, Nano Temper). A sequential titration of unlabeled ligand proteins, PI-PolyPIPosomes, or PI-micelles was made in a Tris-based MST buffer containing 50 mM Tris-HCl, pH 8.0, 50 mM NaCl, 80 mM KCl, and 0.05% Tween-20. This was mixed and loaded into Monolith NT.115 Series capillaries (#MO-K022, Nano Temper), and MST traces were recorded using Monolith NT.115 pico. The binding affinity was automatically generated by MO. Control v1.6 software.

### Immunofluorescence (IF) and confocal microscopy

For immunofluorescence studies, cells were cultured on coverslips coated with 0.2% gelatin (#G9391, MilliporeSigma). The cells were fixed with 4% paraformaldehyde (PFA) (#sc-281692, Santa Cruz Biotechnology) for 20 min at room temperature, followed by three times washing with PBS. Then, the cells were permeabilized with 0.3% Triton-X100 for 10 min and rewashed three times with PBS. Subsequently, the cells were blocked with 3% BSA in PBS for 1 h at room temperature. After blocking, the cells were incubated with primary antibodies overnight at 4 °C. Following primary antibody incubation, the cells were washed three times with PBS and then incubated with fluorescent-conjugated secondary antibodies (Molecular Probes) for 1 h at room temperature. Following secondary antibody incubation, the cells were washed three times with PBS, and the nuclei were counterstained with 1 μg/ml 4′,6-diamidino-2-phenylindole (DAPI) (#D3571, Invitrogen) in PBS for 30 min at room temperature. The cells were rewashed three times with PBS and mounted in Prolong Glass Antifade Mountant media (#P36984, Thermo Fisher Scientific). Leica SP8 3xSTED Super-Resolution Microscope took imaging for high-resolution confocal microscope data. The Leica SP8 3xSTED microscope was controlled by LASX software (Leica Microsystems). All images were acquired using a 100X objective lens (N.A. 1.4 oil). For quantification, the mean fluorescent intensity of channels in each cell was measured by LASX. GraphPad Prism generated the quantitative graph. The images were processed using ImageJ.

### Proximity ligation assay (PLA)

As previously described, PLA detected *in situ* protein-protein/PI interactions (12). After fixation and permeabilization, cells were blocked before incubation with primary antibodies, as in routine IF staining. The cells were then processed for PLA (#DUO92101, MilliporeSigma) according to the manufacturer’s instructions and a previously published protocol. The slides were mounted with Duolink In Situ Mounting Medium with DAPI (#DUO82040, MilliporeSigma). PLA signals were detected using a Leica SP8 confocal microscope, which visualized the signals as discrete punctate foci and provided the intracellular localization of the complex. Quantification of nuclear PLA foci was employed using ImageJ.

### *In vitro* ubiquitination assay

For the *in vitro* ubiquitination assay targeting MDM2 autoubiquitination, 100 nM of E1 enzyme (#E304, R&D Systems), 1 μM of E2 enzyme (#E2627, R&D Systems), 1 μM of MDM2, E3 ligase reaction buffer (#B71, R&D Systems), 10 mM of MgATP solution (#B20, R&D Systems) and 100 μM of ubiquitin (#U100H, R&D Systems) were incubated with different concentrations of αBC, HSP27 (0.9, 1.8, or 2.7 μM), or PIP_2_ (10, 20, or 30 μM). For p53 ubiquitination, 5 μM of p53 was included in the ubiquitination mixture. A negative control was established by replacing MgATP with ddH_2_O. Recombinant MDM2, p53, αBC, and HSP27 were purified, and PIP_2_ was purchased as described above. The ubiquitination mixture was incubated for 1 h at 37 °C, and the reaction was terminated by adding 100 mM of DTT (#R0861, Thermo Fisher Scientific). Subsequently, the ubiquitin conjugation reaction products were separated using SDS–PAGE, and Ubiquitin, MDM2, αBC, and HSP27 were detected by IB. Quantitative analysis was conducted using GraphPad Prism, and images were processed using ImageJ.

### Statistics and reproducibility

Statistical analysis of the data was performed using GraphPad Prism and Microsoft Excel, and data from at least three different experiments were used in this study. Two-tailed unpaired *t*-tests were used for pair-wise significance unless otherwise indicated. We note that no power calculations were used. Sample sizes were determined based on previously published experiments where significant differences were observed. Each experiment was independently repeated at least three times, with the number of repeats defined in each figure legend. We used at least three independent experiments or biologically independent samples for statistical analysis.

## Data availability

Data generated in this study are contained within the article.

## Supporting information

This article contains supporting information Figs. S1–S4.

## Conflict of interest

The authors declare that they have no conflicts of interest with the content of this article.

## References

[1] Mandal K. (2020). Review of PIP2 in cellular signaling, functions and. Dis. Int. J. Mol. Sci..

[2] Choi S., Thapa N., Tan X., Hedman A.C., Anderson R.A. (2015). PIP kinases define PI4,5P_2_signaling specificity by association with effectors. Biochim. Biophys. Acta.

[3] Choi S., Chen M., Cryns V.L., Anderson R.A. (2019). A nuclear phosphoinositide kinase complex regulates p53. Nat. Cell Biol..

[4] Chen M., Wen T., Horn H.T., Chandrahas V.K., Thapa N., Choi S. (2020). The nuclear phosphoinositide response to stress. Cell Cycle.

[5] Mellman D.L., Gonzales M.L., Song C., Barlow C.A., Wang P., Kendziorski C. (2008). A PtdIns4,5P2-regulated nuclear poly(A) polymerase controls expression of select mRNAs. Nature.

[6] Kandoth C., McLellan M.D., Vandin F., Ye K., Niu B., Lu C. (2013). Mutational landscape and significance across 12 major cancer types. Nature.

[7] Feroz W., Sheikh A.M.A. (2020). Exploring the multiple roles of guardian of the genome: P53 Egyptian. J. Med. Hum. Genet..

[8] Walerych D., Lisek K., Sommaggio R., Piazza S., Ciani Y., Dalla E. (2016). Proteasome machinery is instrumental in a common gain-of-function program of the p53 missense mutants in cancer. Nat. Cel. Biol..

[9] Lisek K., Campaner E., Ciani Y., Walerych D., Del Sal G. (2018). Mutant p53 tunes the NRF2-dependent antioxidant response to support survival of cancer cells. Oncotarget.

[10] Olive K.P., Tuveson D.A., Ruhe Z.C., Yin B., Willis N.A., Bronson R.T. (2004). Mutant p53 gain of function in two mouse models of Li-fraumeni syndrome. Cell.

[11] Hanel W., Marchenko N., Xu S., Yu S.X., Weng W., Moll U. (2013). Two hot spot mutant p53 mouse models display differential gain of function in tumorigenesis. Cell Death Differ..

[12] Chen M., Choi S., Wen T., Chen C., Thapa N., Lee J.H. (2022). A p53–phosphoinositide signalosome regulates nuclear AKT activation. Nat. Cel. Biol..

[13] Honda R., Yasuda H. (2000). Activity of MDM2, a ubiquitin ligase, toward p53 or itself is dependent on the RING finger domain of the ligase. Oncogene.

[14] Fang S., Jensen J.P., Ludwig R.L., Vousden K.H., Weissman A.M. (2000). Mdm2 is a RING finger-dependent ubiquitin protein ligase for itself and p53∗. J. Biol. Chem..

[15] Peng Y., Chen L., Li C., Lu W., Chen J. (2001). Inhibition of MDM2 by hsp90 contributes to mutant p53 stabilization∗. J. Biol. Chem..

[16] Wiech M., Olszewski M.B., Tracz-Gaszewska Z., Wawrzynow B., Zylicz M., Zylicz A. (2012). Molecular mechanism of mutant p53 stabilization: the role of HSP70 and MDM2. PloS One.

[17] Yang L., Song T., Cheng Q., Chen L., Chen J. (2019). Mutant p53 sequestration of the MDM2 acidic domain inhibits E3 ligase activity. Mol. Cell. Biol..

[18] Lewis A.E., Sommer L., Arntzen M., Strahm Y., Morrice N.A., Divecha N. (2011). Identification of nuclear phosphatidylinositol 4,5-bisphosphate-interacting proteins by neomycin extraction. Mol. Cell Proteomics.

[19] Choi S., Thapa N., Hedman A.C., Li Z., Sacks D.B., Anderson R.A. (2013). IQGAP1 is a novel phosphatidylinositol 4,5 bisphosphate effector in regulation of directional cell migration. EMBO J..

[20] de Queiroz R.M., Efe G., Guzman A., Hashimoto N., Kawashima Y., Tanaka T. (2024). Mdm2 requires Sprouty4 to regulate focal adhesion formation and metastasis independent of p53. Nat. Commun..

[21] Barlow C.A., Laishram R.S., Anderson R.A. (2010). Nuclear phosphoinositides: a signaling enigma wrapped in a compartmental conundrum. Trends Cell Biology.

[22] Adhikari A.S., Sridhar Rao K., Rangaraj N., Parnaik V.K., Mohan Rao C. (2004). Heat stress-induced localization of small heat shock proteins in mouse myoblasts: intranuclear lamin A/C speckles as target for αB-crystallin and Hsp25. Exp. Cell Res..

[23] Bryantsev A.L., Kurchashova S.Y., Golyshev S.A., Polyakov V.Y., Wunderink H.F., Kanon B. (2007). Regulation of stress-induced intracellular sorting and chaperone function of Hsp27 (HspB1) in mammalian cells. Biochem. J..

[24] Watanabe G., Kato S., Nakata H., Ishida T., Ohuchi N., Ishioka C. (2009). αB-crystallin: a novel p53-target gene required for p53-dependent apoptosis. Cancer Sci..

[25] Sohlenkamp C., López-Lara I.M., Geiger O. (2003). Biosynthesis of phosphatidylcholine in bacteria. Prog. Lipid Res..

[26] Lorick K.L., Jensen J.P., Fang S., Ong A.M., Hatakeyama S., Weissman A.M. (1999). RING fingers mediate ubiquitin-conjugating enzyme (E2)-dependent ubiquitination. Proc. Natl. Acad. Sci. U. S. A..

[27] Minsky N., Oren M. (2004). The RING domain of Mdm2 mediates histone ubiquitylation and transcriptional repression. Mol. Cell.

[28] Yang J.-Y., Zong C.S., Xia W., Yamaguchi H., Ding Q., Xie X. (2008). ERK promotes tumorigenesis by inhibiting FOXO3a via MDM2-mediated degradation. Nat. Cel. Biol..

[29] Boronenkov I.V., Loijens J.C., Umeda M., Anderson R.A. (1998). Phosphoinositide signaling pathways in nuclei are associated with nuclear speckles containing pre-mRNA processing factors. Mol. Biol. Cell.

[30] Ahn J.Y., Liu X., Cheng D., Peng J., Chan P.K., Wade P.A. (2005). Nucleophosmin/B23, a nuclear PI(3,4,5)P(3) receptor, mediates the antiapoptotic actions of NGF by inhibiting CAD. Mol. Cel..

[31] Jones D.R., Bultsma Y., Keune W.J., Halstead J.R., Elouarrat D., Mohammed S. (2006). Nuclear PtdIns5P as a transducer of stress signaling: an in vivo role for PIP4Kbeta. Mol. Cel..

[32] Viiri K.M., Jänis J., Siggers T., Heinonen T.Y., Valjakka J., Bulyk M.L. (2009). DNA-binding and -bending activities of SAP30L and SAP30 are mediated by a zinc-dependent module and monophosphoinositides. Mol. Cell. Biol..

[33] Bua D.J., Martin G.M., Binda O., Gozani O. (2013). Nuclear phosphatidylinositol-5-phosphate regulates ING2 stability at discrete chromatin targets in response to DNA damage. Sci. Rep..

[34] Gelato Kathy A., Tauber M., Ong Michelle S., Winter S., Hiragami-Hamada K., Sindlinger J. (2014). Accessibility of different histone H3-binding domains of UHRF1 is allosterically regulated by phosphatidylinositol 5-phosphate. Mol. Cel..

[35] Blind R.D., Sablin E.P., Kuchenbecker K.M., Chiu H.J., Deacon A.M., Das D. (2014). The signaling phospholipid PIP3 creates a new interaction surface on the nuclear receptor SF-1. Proc. Natl. Acad. Sci. U. S. A..

[36] Bieging K.T., Mello S.S., Attardi L.D. (2014). Unravelling mechanisms of p53-mediated tumour suppression. Nat. Rev. Cancer.

[37] Fukami K., Matsuoka K., Nakanishi O., Yamakawa A., Kawai S., Takenawa T. (1988). Antibody to phosphatidylinositol 4,5-bisphosphate inhibits oncogene-induced mitogenesis. Proc. Natl. Acad. Sci. U. S. A..

[38] Fukami K., Endo T., Imamura M., Takenawa T. (1994). alpha-Actinin and vinculin are PIP2-binding proteins involved in signaling by tyrosine kinase. J. Biol. Chem..

[39] Bakthisaran R., Tangirala R., Rao C.M. (2015). Small heat shock proteins: role in cellular functions and pathology. Biochim. Biophys. Acta (BBA) - Proteins Proteomics.

[40] van den Ijssel P., Wheelock R., Prescott A., Russell P., Quinlan R.A. (2003). Nuclear speckle localisation of the small heat shock protein αB-crystallin and its inhibition by the R120G cardiomyopathy-linked mutation. Exp. Cell Res..

[41] Xu Z., Wu W., Yan H., Hu Y., He Q., Luo P. (2021). Regulation of p53 stability as a therapeutic strategy for cancer. Biochem. Pharmacol..

[42] Alexandrova E.M., Yallowitz A.R., Li D., Xu S., Schulz R., Proia D.A. (2015). Improving survival by exploiting tumour dependence on stabilized mutant p53 for treatment. Nature.

[43] Mantovani F., Collavin L., Del Sal G. (2019). Mutant p53 as a guardian of the cancer cell. Cell Death Differ..

[44] den Engelsman J., Keijsers V., de Jong W.W., Boelens W.C. (2003). The small heat-shock protein αB-crystallin promotes FBX4-dependent ubiquitination. J. Biol. Chem..

[45] Parcellier A., Schmitt E., Gurbuxani S., Seigneurin-Berny D., Pance A., Chantôme A. (2003). HSP27 is a ubiquitin-binding protein involved in I-kappaBalpha proteasomal degradation. Mol. Cell. Biol..

[46] Parcellier A., Brunet M., Schmitt E., Col E., Didelot C., Hammann A. (2006). HSP27 favors ubiquitination and proteasomal degradation of p27Kip1 and helps S-phase re-entry in stressed cells. FASEB J..

[47] Kumano M., Furukawa J., Shiota M., Zardan A., Zhang F., Beraldi E. (2012). Cotargeting stress-activated Hsp27 and autophagy as a combinatorial strategy to amplify endoplasmic reticular stress in prostate cancer. Mol. Cancer Ther..

[48] Vahid S., Thaper D., Gibson K.F., Bishop J.L., Zoubeidi A. (2016). Molecular chaperone Hsp27 regulates the Hippo tumor suppressor pathway in cancer. Sci. Rep..

[49] Wen T., Chen M., Cryns V.L., Anderson R.A. (2025). The poly(A) polymerase star-PAP is regulated by stably associated phosphoinositide messengers. J. Biol. Chem..

[50] Chen C., Carrillo N.D., Chen M., Wen T., Awasthi P., Anderson R.A. (2025). Regulation of NRF2 by stably associated phosphoinositides and small heat shock proteins in response to stress. J. Biol. Chem..

