# Supplementary material for: Regulation of the MDM2-p53 nexus by a nuclear phosphoinositide and small heat shock protein complex
Source: J Biol Chem. 2025 Jul 26;301(9):110527. doi: 10.1016/j.jbc.2025.110527 (PMC12409426; doi:10.1016/j.jbc.2025.110527)
Supplement: Supporting Figures [file mmc1.docx]

**Supporting Information for**

Regulation of the MDM2-p53 Nexus by a Nuclear Phosphoinositide and Small Heat Shock Protein Complex

Jeong Hyo Lee, Mo Chen, Tianmu Wen, Richard A. Anderson^*^, and Vincent L. Cryns^*^

*Correspondence: Richard A. Anderson and Vincent L. Cryns

**Email:**  raanders@wisc.edu (R.A.A.); vlcryns@medicine.wisc.edu (V.L.C.)

**This PDF file includes:**

Figures S1 to S4 and the accompanying figure legends.

Supporting Information

**
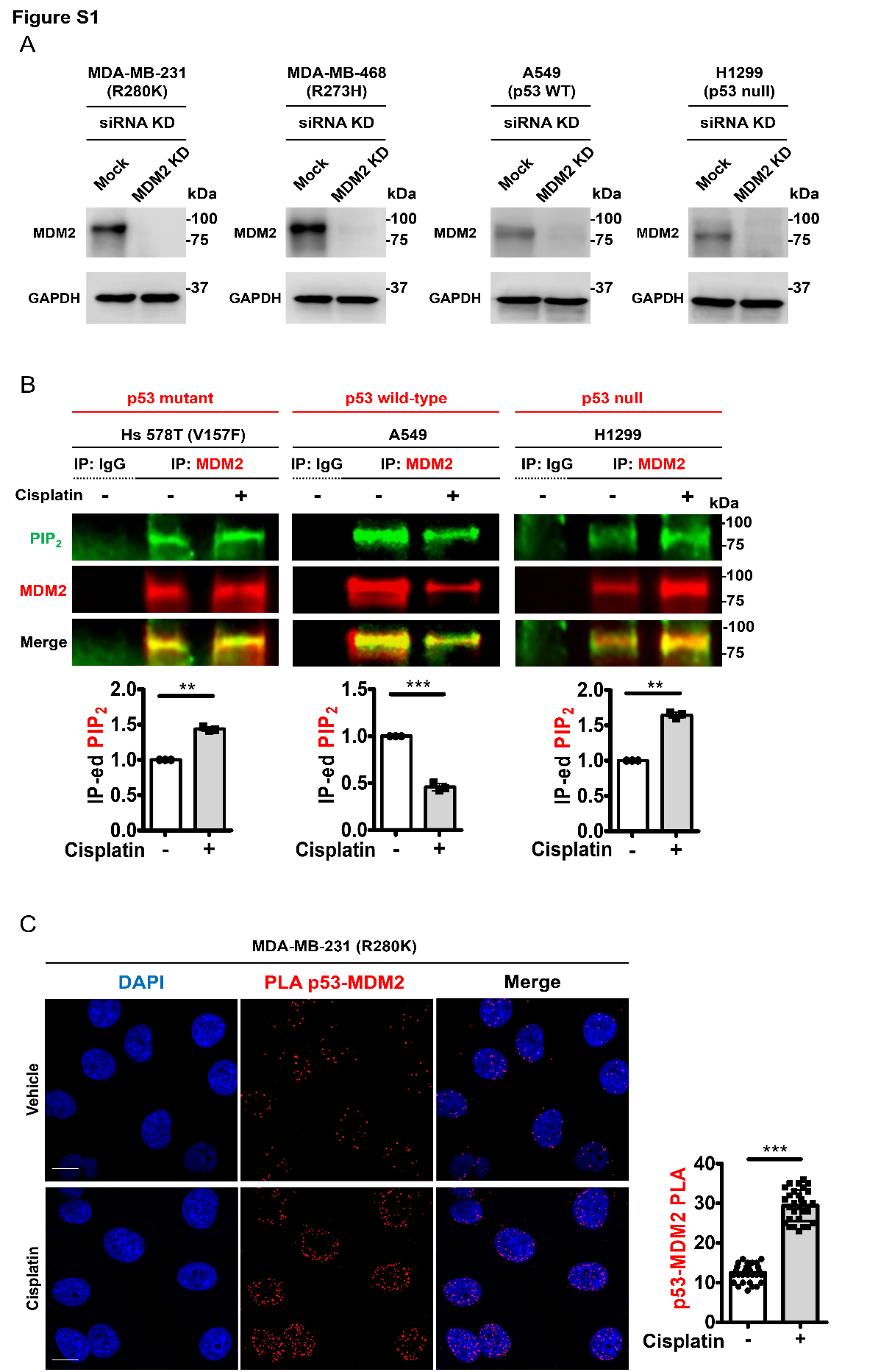
**

**Figure S1: MDM2 stably associates with PIP_2_ in multiple cell lines**

***A*,** MDA-MB-231, MDA-MB-468, A549, and H1299 cells were transfected with siRNAs targeting MDM2. After 48 h, IB analyzed endogenous MDM2. KD, knockdown. The experiments were repeated three times.

***B***, HS578T, A549, and H1299 cells were treated with 30 µM cisplatin or vehicle for 24 h, then processed for IP of MDM2 and fluorescence IB. Fluorescence IP−IB detects stress-induced PIP_2_ association with endogenous MDM2.  The PIP_2_ IB intensity was quantified, and the graph is shown as mean ± s.d. of *n* = 3 independent experiments.

***C***, PLA of MDM2-p53 in MDA-MB-231 cells treated with vehicle or 30 µM cisplatin for 24 h. The nuclear PLA foci of MDM2-p53 were quantified. *n* = 30 cells pooled from 3 independent experiments, 10 cells per experiment.


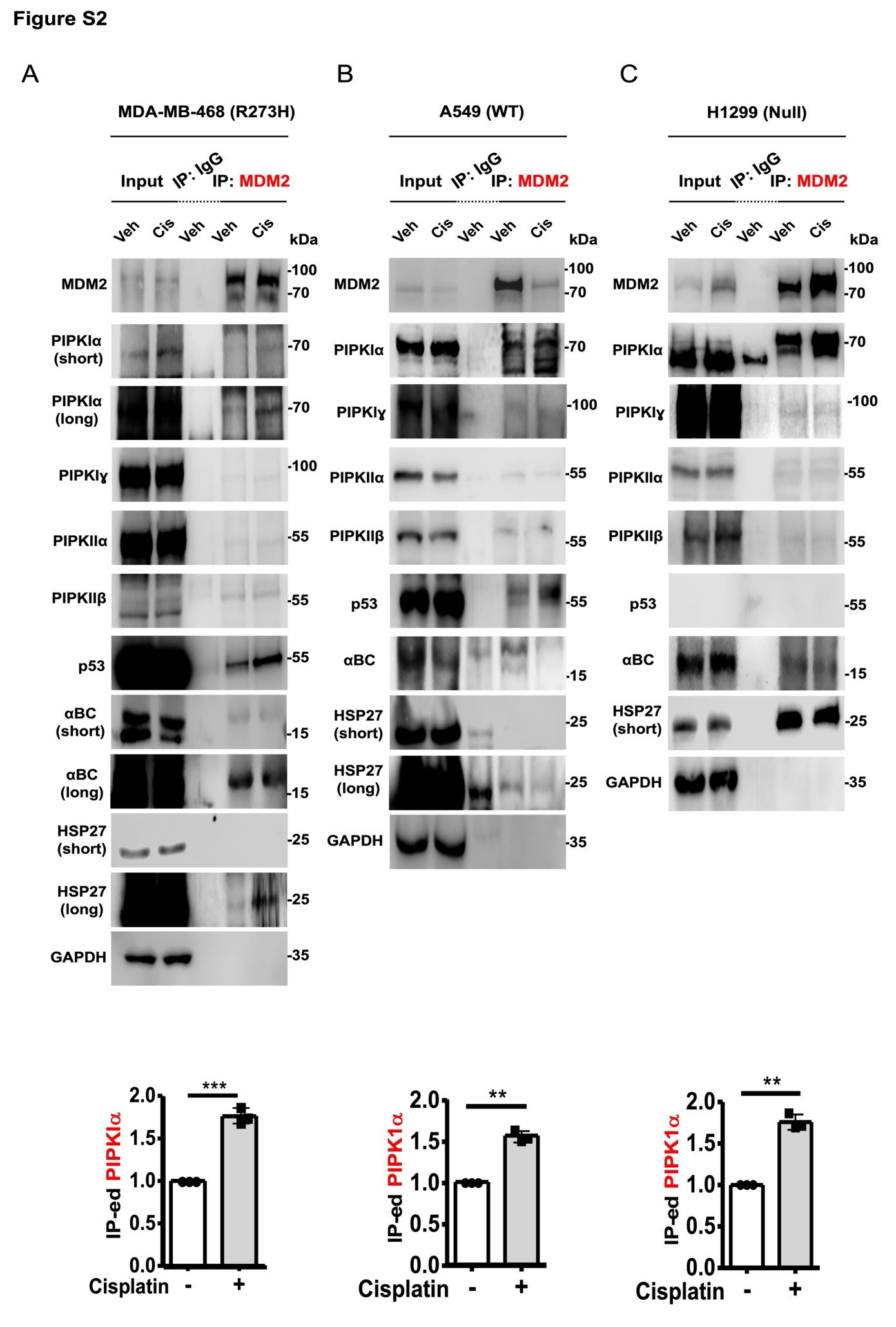


**Figure S2: MDM2 associates with PIP kinases and sHSPs in various cell lines**

***A*-*C*,** Co-IP of endogenous MDM2 from MDA-MB-468 (*A*), A549 (*B*), and H1299 (*C*) cells treated with vehicle or 30 µM cisplatin for 24 h. The MDM2, PIPKIα, PIPKIγ, PIPKIIα, PIPKIIβ, p53, αBC, HSP27 and GAPDH co-IPed by MDM2 were analyzed by IB. Veh, vehicle; Cis, cisplatin-treated, short, short-time exposure; long, long-time exposure.


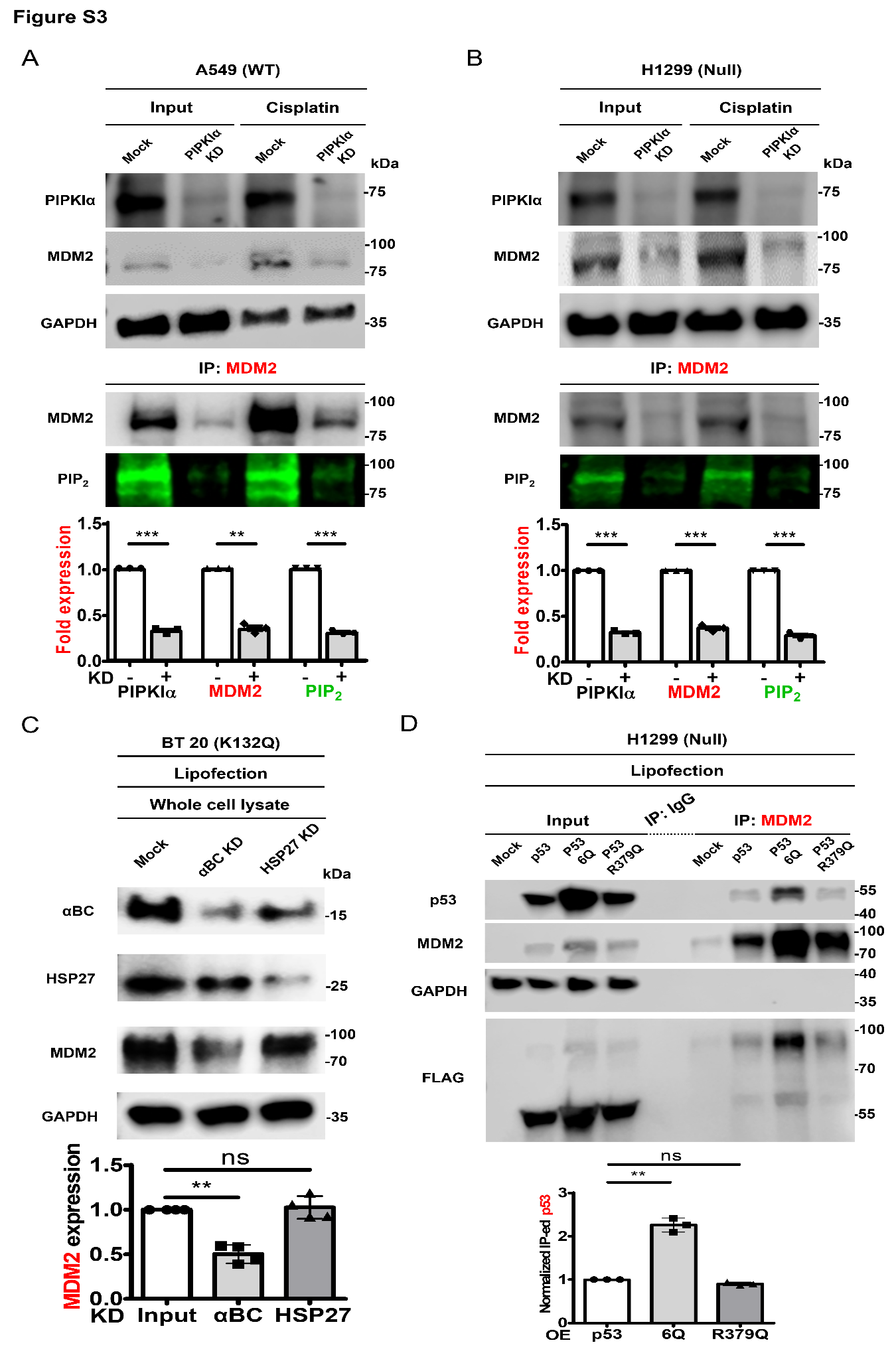


**Figure S3: PIPKIα and sHSP KD affect MDM2 stability**

***A*, *B*,** A549 (*A*), and H1299 cells (*B*) were transfected with siRNAs targeting PIPKIα. After 24 h, cells were treated with 30 µM cisplatin for an additional 24 h. IB analyzed the expression of the indicated proteins. The IBs of PIPKIα, MDM2, and PIP_2_ were quantified. The graph shows mean ± s.d. of n = 3 independent experiments. KD, knockdown.

***C***, BT20 cells were transfected with siRNAs for αBC or HSP27. Expression of αBC, HSP27, MDM2, and GAPDH was analyzed by IB, and αBC and HSP27 IBs were quantified. The graph shows mean ± s.d. of n = 3 independent experiments. KD, knockdown. KD, knockdown.

***D***, H1299 cells were transfected with wild-type p53, p53 6Q or p53 R379 mutant for 48 h. Empty vector (Mock) was used as a negative control. Cells were subjected to Co-IP with MDM2. The expression of the indicated proteins, MDM2, p53, GAPDH, and FLAG tag, was analyzed by IB and IPed p53 level was normalized with MDM2 expression then quantified. The graph shows mean ± s.d. of n = 3 independent experiments.

**
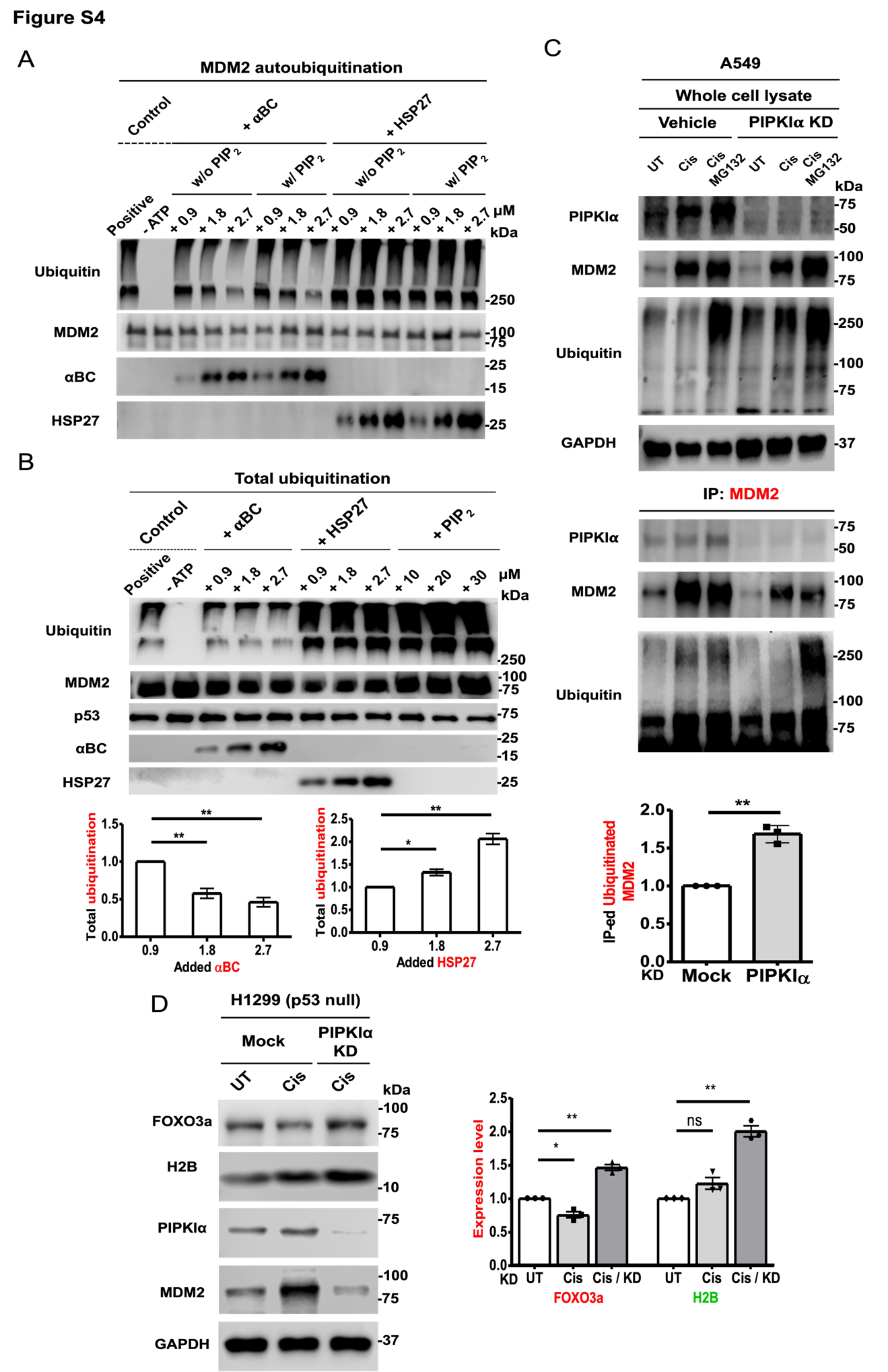
**

**Figure S4: Regulation of MDM2 ubiquitination and protein levels by αBC and PIPKIα**

***A***, For MDM2 autoubiquitination, 100 nM of E1 enzyme, 1 μM of E2 enzyme, 1 μM of MDM2, E3 ligase reaction buffer, 10mM of MgATP solution, and 100 μM of ubiquitin were incubated with different concentrations of αBC and HSP27 (0.9, 1.8, or 2.7 μM) in the absence or presence of PIP_2_ (10 μM) for 1 h. IB analyzed Ubiquitin, MDM2, αBC, and HSP27. The experiments were repeated three times.

***B***, For p53 in vitro ubiquitination, 100 nM of E1 enzyme, 1 μM of E2 enzyme, 1 μM of MDM2, E3 ligase reaction buffer, 1 μM of p53, 110mM of MgATP solution and 100 μM of ubiquitin were incubated with different concentrations of αBC, HSP27 (0.9, 1.8, or 2.7 μM), or PIP_2_ (10, 20, or 30 μM) for 1 h. Ubiquitin, MDM2, p53, αBC and HSP27 were analyzed by IB, and ubiquitin IBs were quantified. The graphs are shown as mean ± s.d. of *n* = 3 independent experiments.

***C***, A549 cells were transfected with siRNAs for PIPKIα and treated with vehicle or 30 µM cisplatin for 24 h. After cells were treated with 10 μM of MG132 for 4 h, cells were harvested for IP of MDM2. IB analyzed the expression of the indicated proteins, and ubiquitin IBs were quantified. The graph shows mean ± s.d. of n = 3 independent experiments. KD, knockdown; UT, untreated; Cis, cisplatin-treated; Cis/MG132, Cisplatin/MG132 treated.

***D***, H1299 cells were transfected with siRNAs for PIPKIα and treated with vehicle or 30 µM cisplatin for 24 h. After cells were harvested for IB. The expression of the indicated proteins was analyzed and quantified. The graph shows mean ± s.d. of n = 3 independent experiments. Mock, empty vector; KD, knockdown; UT, untreated; Cis, cisplatin-treated.
